# The Relationship Between Aldose Reductase and Isoxazole Derivatives: An In Vitro and In Silico Approach to Its Correlation With Diabetic Conditions

**DOI:** 10.1002/bab.70003

**Published:** 2025-06-06

**Authors:** Ahmet Esat Göner, Hatice Esra Duran

**Affiliations:** ^1^ Department of Medical Biochemistry, Faculty of Medicine Kafkas University Kars Turkey

**Keywords:** aldose reductase, diabetic complications, inhibition, in silico study, isoxazole

## Abstract

Diabetes mellitus (DM), which can result in a number of problems such as cataracts, neuropathy, retinopathy, nephropathy, and several cardiovascular illnesses, continues to be a growing issue despite major advancements in treatment approaches. Numerous scientists have targeted the polyol pathway as a target for intervention since it includes aldose reductase (ALR2, AR (E.C.1.1.1.21)), a crucial enzyme. Oxidative damage, NADPH depletion, and intracellular sorbitol buildup result from the overactivation of ALR2 brought on by hyperglycemia. Interest in creating novel ALR2 inhibitors (ALR2Is) with enhanced therapeutic characteristics has increased as a result of this circumstance. The amazing biological capabilities of isoxazole molecules led us to look into the biological properties of isoxazole and related compounds. We examined these isoxazoles' binding affinities and interactions in the ALR2 active site using thorough in vitro and in silico techniques. In comparison to the reference pharmaceutical epalrestat (EPR, *K*_I_ 232.70 ± 15.51 nM), our results demonstrate that these isoxazoles efficiently inhibit ALR2 at nanomolar doses, with inhibition constants (*K*_I_) ranging from 12.13 ± 1.24 nM to 89.51 ± 4.68 nM. Important interactions between these isoxazoles and ALR2 are highlighted by the combined in vitro and in silico studies, indicating their potential as therapeutic agents against a range of pathological diseases. Furthermore, these substances that have ALR2 inhibitory properties could be useful as stand‐in treatments or preventative measures for diabetes problems.

## 1 Introduction

Aldose reductase (AR, ALR2, AKR1B1; EC 1.1.1.21) is indeed a crucial enzyme that belongs to the aldo‐keto reductase (AKR) superfamily [1]. This family of enzymes is responsible for the reduction of a wide variety of aldehydes and ketones to their corresponding alcohols, using NADPH as a cofactor [2]. Its function is to catalyze the reduction of several harmful aldehydic substrates to their corresponding, less toxic alcohols. The reduction of biogenic aldehydes produced by the breakdown of catecholamines, phospholipids, and steroids is catalyzed by the ALR2 enzyme. Consequently, ALR2 functions in numerous organs as an extrahepatic detoxifying enzyme [3].

**FIGURE 1:**
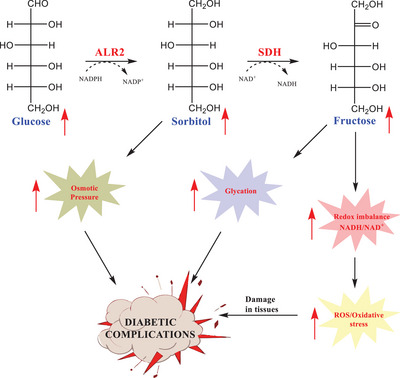
Polyol pathway [9].

Aldose reductase plays a significant role in the polyol pathway, where it catalyzes the reduction of glucose to sorbitol [4]. Under normal physiological conditions, in glycolysis, the enzyme hexokinase phosphorylates the glucose molecule, converting it into glucose‐6‐phosphate. However, due to the low affinity of the enzyme aldose reductase for glucose under these conditions, about 3% of the unphosphorylated glucose enters the polyol pathway, where it is metabolized into sorbitol and fructose [5]. However, in hyperglycemic states, such as diabetes, the activity of aldose reductase significantly increases in various tissues [6]. As a result, when the hexokinase enzyme becomes saturated, up to 33% of the total glucose is diverted into the polyol pathway. This heightened involvement of aldose reductase in glucose metabolism contributes significantly to the development of diabetic complications such as neuropathy, retinopathy, and cataracts [7, 8] (Figure 1).

**FIGURE 2:**
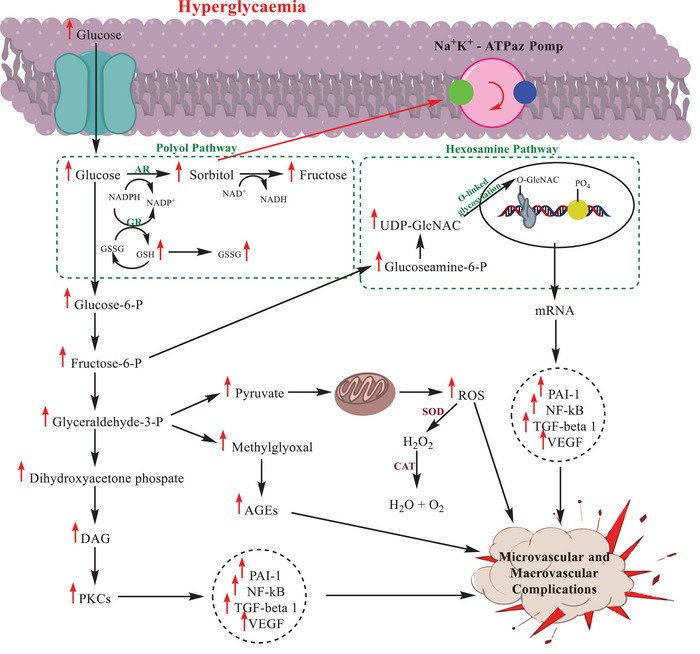
Diagrammatic illustration of the glucose metabolism routes, including the polyol, hexosamine, and PKC pathways, in the development of diabetes problems. The list of abbreviations is as follows: AGEs: advanced glycation end products, AR: aldose reductase, CAT: catalase, DAG: diacylglycerol, Fructose‐6‐P: fructose‐6‐phosphate, Glucose‐6‐P: glucose‐6‐phosphate, Glyceraldehyde‐3‐P: glyceraldehyde‐3‐phosphate, GR: glutathione reductase, NF‐κB: nuclear factor kappa B, PAI‐1: plasminogen activator inhibitor‐1, PARP: poly (ADP‐ribose) polymerase, PKC: protein kinase C, SOD: superoxide dismutase, TGF‐beta 1: transforming growth factor‐beta 1, UDP‐GlcNAC: uridine diphosphate *N*‐acetylglucosamine, VEGF: vascular endothelial growth factor [20].

Under normoglycemic circumstances, ALR2's affinity for glucose is minimal [10] and the majority of glucose is present as glucose‐6‐phosphate and is subjected to glycolysis [11, 12]. Because phosphorylated species have a low affinity for ALR2, only a small fraction of unphosphorylated glucose can enter the polyol pathway [13, 14, 15]. In contrast, increased blood glucose during a hyperglycemic state causes accelerated transport through the polyol pathway, which leads to the intracellular buildup of sorbitol, a depletion of cytoplasmic NADPH, oxidative stress, and decreased glutathione levels [16] (Figure 2). The relationship among ALR2 and issues associated with diabetes has motivated efforts to create ALR2 inhibitors as therapies; a number of these medications have been found from natural sources and developed synthetically [17, 18, 19].

According to predictions from the World Health Organization (WHO), there are 580 million diabetics globally, and by 2030, there should be at least 643 million [21]. In recent decades, organic substances have become more and more popular for both healing and avoiding Type 2 diabetes. Medicinal plants, including infusions of herbs and supplements, have been used to treat a variety of conditions, including diabetes [22]. Many more naturally occurring ALR2Is, including monoterpenes, stilbenes, coumarins, flavonoids, and related aromatic chemicals, have been documented in the literature [23, 24]. It has been demonstrated that flavonoids from fruits, vegetables, herbs, and spices inhibit ALR2 activity [25]. There is evidence that a number of natural compounds, such as flavonoids, phenolics, alkaloids, and terpenoids, may have anti‐diabetic properties. Certain flavonoids, polyphenols, and sugar compounds proved successful in blocking aldose reductase's inhibitory action [26, 27, 28].

*N*‐containing heterocyclic compounds are a type of significant heterocyclic compounds that are frequently employed in the quest for new bioactive chemicals and play an essential role in the domains of medical chemistry [29]. One type of heterocyclic chemical that is frequently employed in drug discovery research is isoxazole [30]. Isoxazole is indeed a crucial pharmacophore, [29, 31–32] widely recognized for its versatile chemical structure—a five‐membered heterocyclic ring composed of three carbon atoms, one oxygen, and one nitrogen. This unique structure imparts isoxazole with a range of chemical and biological activities, making it a valuable core in drug development, agrochemicals, and materials science. Dozens of medications that include isoxazole fragments are currently on the market. These medications can prevent and treat a wide range of illnesses, protecting people's health in the process [33].

The high reactivity of isoxazoles, particularly in basic environments, stems from the electron‐rich nature of the ring, which allows for easy participation in nucleophilic reactions. This reactivity makes it synthetically accessible, allowing for numerous derivatizations and functionalizations that can modulate its properties, such as increasing its bioavailability, specificity, or stability in pharmaceutical applications. Recent advancements in the synthesis of isoxazoles have expanded the scope of their utility. Methods such as cycloadditions, metal‐catalyzed reactions, and multi‐component reactions have streamlined their production and enabled the introduction of functional groups at different positions of the ring, broadening the potential for new applications. These advancements have opened doors to developing novel isoxazole‐based drugs with potent antimicrobial, anti‐inflammatory, and anticancer activities. The ongoing research into isoxazole derivatives continues to highlight their role as a key scaffold in medicinal chemistry due to their favorable pharmacokinetic properties and the possibility of diverse chemical modifications [34].

The enzyme's structure, with a typical (α/β)8‐barrel fold characteristic of the AKR superfamily, allows for binding interactions with substrates and inhibitors within its catalytic pocket. Molecular docking studies on potential ALR2Is, such as those involving isoxazole derivatives, seek to exploit this binding pocket to design compounds that can effectively inhibit ALR2 activity. The inhibition of ALR2 is considered a promising strategy for managing and potentially preventing the progression of diabetes‐related complications [35].

**FIGURE 3:**
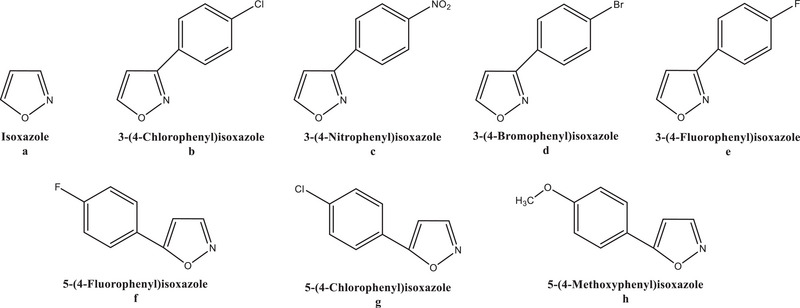
The chemical structures of the isoxazoles that were employed in this investigation.

In this study, the isoxazole scaffold, due to its structural versatility and ability to interact with various biological targets, has been explored as a potential ALR2I (Figure 3). Molecular docking studies, which involve computational simulations to predict the binding of a molecule to a target protein, have provided valuable insights into the interaction of isoxazoles with aldose reductase. These studies help identify key binding sites and interactions between the isoxazole core and the enzyme's active site, allowing researchers to design more potent and selective inhibitors.

## 2 Materials and Methods

### 2.1 Chemicals

All of the compounds utilized in the investigation were purchased from Sigma–Aldrich.

### 2.2 ALR2 Enzyme Activity Measurement

By tracking the shift in absorbance at 340 nm brought on by a decrease in NADPH, ALR2 activity was ascertained. The reaction mixture for ALR2 activity consists of 0.8 M Na‐phosphate buffer (pH = 5.5), NADPH (0.11 mM), DL‐glyceraldehyde (4.7 mM), and enzyme solution [36, 37]. The reference molecule employed was epalrestat (EPR) (Figure 3). Every measurement was taken three times.

### 2.3 In Vitro Inhibition Studies

**FIGURE 4:**
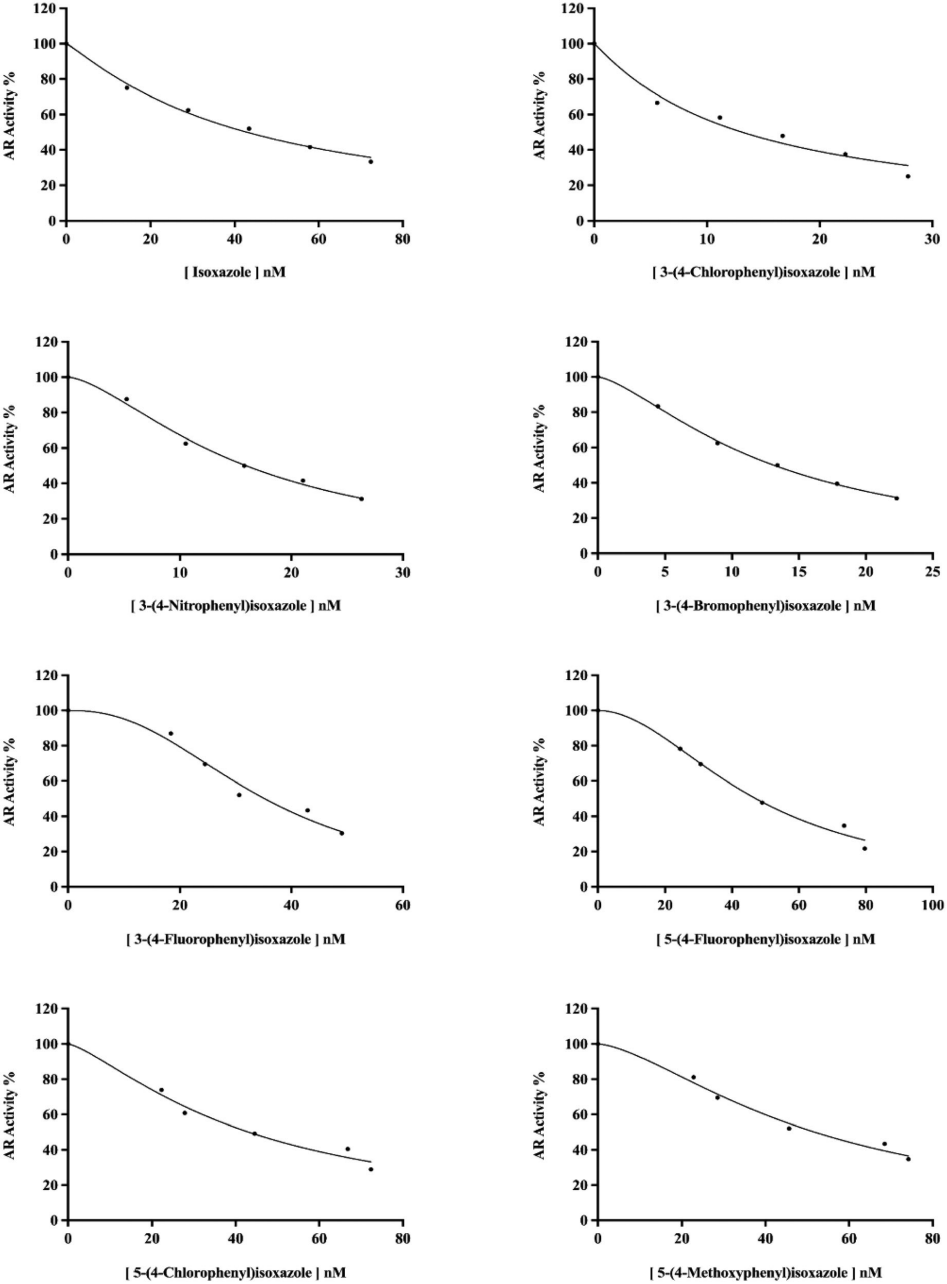
IC_50_ graphs of isoxazole and its derivatives on the ALR2 enzyme.

**FIGURE 5:**
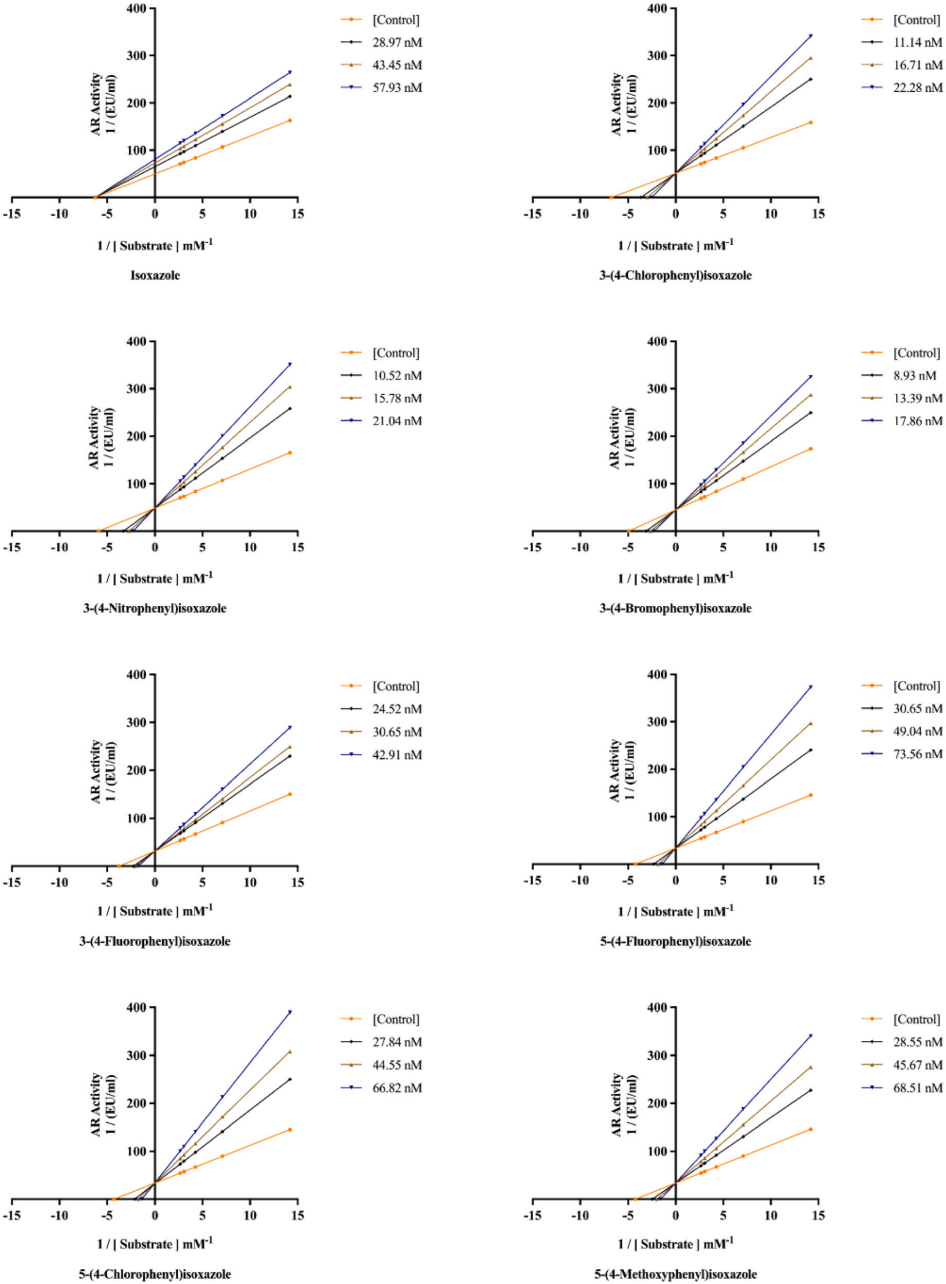
Lineweaver–Burk plots of isoxazole and its derivatives on the ALR2 enzyme.

Isoxazole and its derivatives' inhibitory effects on ALR2 were evaluated at least five distinct inhibitor concentrations. Similar to the previous studies [38, 39, 40, 41], we used the Activity (%) − (Compound) graphs (Figure 4) for each derivative to calculate the IC_50_ values of the isoxazoles. The *K*_I_ values and inhibition types were ascertained using the Lineweaver and Burk curves [42, 43, 44, 45, 46, 47] (Figure 5).

### 2.4 In Silico Study

This molecular docking investigation employed a comprehensive in silico approach using the 2024‐3 version of the Schrödinger Small‐Molecule Drug Discovery Suite, a robust platform compatible with Mac systems and designed for detailed ligand‐protein interaction modeling. The high‐resolution crystallographic data of ALR2 was retrieved from the Research Collaboratory for Structural Bioinformatics Protein Data Bank (RCSB PDB), utilizing PDB entry 4JIR [48, 49, 50, 51]. This structure provided the template frameworks for analyzing ligand interactions with this enzyme.

In preparation for docking, the protein structure underwent refinement and adjustment using the Protein Preparation Wizard within the Schrödinger Suite [50, 51]. This process involved several critical steps to optimize the accuracy of subsequent ligand‐binding simulations. Initially, all non‐essential water molecules were removed from the crystallographic data to minimize steric clashes within the active site, ensuring a focused binding environment. Hydrogen atoms were added to stabilize polar groups, followed by ionization state optimization of amino acid residues at physiological pH to reflect the proteins' natural state in biological systems. In addition, specific side chains near the active site were assessed for orientation and protonation adjustments to accurately model hydrogen bonding and other electrostatic interactions relevant to ligand binding [52].

The ligands used in this study, namely the isoxazole and its derivatives (a–h), were initially designed in two‐dimensional structures using ChemDraw version 21 (PerkinElmer, Inc., Waltham, MA, USA) for Mac. To enable accurate three‐dimensional docking, the LigPrep module [53, 54] was employed to optimize each compound's molecular geometry, employing the OPLS4 (Optimized Potentials for Liquid Simulations 4) force field for energy minimization [55, 56]. This procedure also included a protonation state adjustment, refined by the Epik tool, which adjusted ionization states for each ligand to physiological pH (7.0) [57, 58, 59]. Epik's functionality in handling multiple tautomeric and protonation states ensured a realistic representation of each ligand's binding conformation, critical for accurate molecular interaction predictions [49, 59].

The potential ligand‐binding region was identified within ALR2 using SiteMap software, a module designed to detect pockets that exhibit optimal physicochemical properties for ligand accommodation. SiteMap identified critical residues within the active site of this enzyme [60, 61], providing insights into the steric and electronic compatibility of the derivatives with these sites. These active site predictions were then incorporated into the Receptor Grid Generation module to create customized receptor grids centered around the binding sites, enhancing the precision of the docking study [62, 63].

Docking simulations were conducted using the Glide module, with the extra precision (XP) docking protocol [64, 65, 66], chosen for its enhanced ability to predict high‐selectivity interactions within active site regions. The XP mode optimizes docking by rigorously scoring each ligand pose according to its affinity for the active site, refining the position of each docked ligand to capture subtle hydrophobic and hydrophilic interactions. Post‐docking, each ligand's conformational state was refined via post‐docking minimization, maximizing the stability of ligand‐enzyme interactions and reducing potential energy discrepancies due to minor positional shifts.

To complement the docking analysis, the pharmacokinetic properties of the isoxazole and its derivatives (a–h) were projected using QikProp software, [52, 67–69] which evaluates absorption, distribution, metabolism, and excretion (ADME) properties. By analyzing the ADME profiles, this tool offered insights into each ligand's drug‐likeness, bioavailability, and overall suitability as a therapeutic agent, providing crucial data on each compound's potential as an ALR2 inhibitor (Table 2).

### 2.5 Statistical Studies

For data analysis and graph creation, GraphPad Prism version 8 for Mac was utilized (GraphPad Software, La Jolla, California, USA). Utilizing SigmaPlot version 12 for Windows (Systat Software, San Jose, California, USA), the inhibition constants were ascertained. To assess how well the enzyme inhibition models matched one another, the extra sum‐of‐squares *F* test and the AICc technique were utilized. The results were presented as the standard error of the mean plus a 95% confidence interval around the mean. A difference between two datasets was considered statistically significant when the *p* value was less than 0.05.

## 3 Results and Discussion

### 3.1 Biological Evaluation

Globally, diabetes mellitus (DM) is becoming more common and is currently a serious health concern for individuals [69, 70]. Type II diabetes mellitus, or T2DM, is a metabolic hormonal disorder that is not dependent on insulin. It is characterized by hyperglycemia, which is brought on by irregularities in insulin secretion, insulin resistance (IR) in target tissues, increased hepatic glucose production, and decreased tissue glucose uptake [71, 72]. The heightened rate of glucose synthesis in the liver is substantially linked to the amounts of enzymes involved in the metabolism of carbohydrates, including glucose‐6‐phosphatase (G6Pase), phosphoenol pyruvate carboxy kinase (PEPCK), and AMP‐activated protein kinase (AMPK) [73, 74, 75]. In order to prevent chronic diabetes, one of the most crucial strategies is to either activate or obstruct the expression of the enzyme.

In the field of medical chemistry, heterocyclic molecules are essential. Because of their numerous biological actions, nitrogen‐containing heterocyclic compounds in particular, the isoxazole scaffold are highly significant among them. Alkaloids with substituted isoxazole groups have antibacterial, anti‐inflammatory, antioxidant, antitubercular, anticancer, analgesic, and GABA (γ‐aminobutyric acid) agonist properties [76]. One neighboring oxygen and nitrogen atom and a five‐membered ring make up the heterocyclic molecule isoxazole [29]. It is commonly recognized that the main pharmacophore area in the structure of bioactive chemicals that is responsible for bioactivity is the isoxazole ring. The hydrogen bond interactions that the electronegative heteroatoms at the 1,2‐position generated with enzymes and receptors are assumed to be the source of the pharmacophoric impact of the isoxazole ring [77].

In accordance with this knowledge, the effects of isoxazole and several of its derivatives on the ALR2 enzyme were investigated in vitro through comparison with the reference medicine EPR. To assess the relative potency of compounds (a–h) and EPR (positive control), the *K*_I_ values were calculated (Table 1). Compounds a–h exhibited ALR2 suppression, according to the in vitro results, with *K*_I_ values ranging from 12.13 ± 1.24 to 89.51 ± 4.68 nM. It was discovered that compounds an inhibited ALR2 as noncompetitive, whereas compounds b–h acted as competitive ALR2 inhibitors. Based on their *K*_I_ values, compounds a–h were arranged in the following order, going from most active to least active: compound c; compound b; compound d; compound g; compound h; compound e; compound f; compound a.

**TABLE 1:** IC_50_ and *K*_I_ values and inhibition types of isoxazole and its derivatives (a–h) on ALR2 enzyme.

Compound		IC_50_ ^*^ (nM)	*R*2	*K*_I_ ^*^ (nM)	*R*2	Inhibition type
ID	Name					
**a**	Isoxazole	43.00 ± 1.64	0.9935	89.51 ± 4.68	0.9890	Noncompetitive
**b**	3‐(4‐chlorophenyl) isoxazole	13.14 ± 1.20	0.9727	12.90 ± 1.39	0.9836	Competitive
**c**	3‐(4‐nitrophenyl) isoxazole	15.96 ± 0.50	0.9939	12.13 ± 1.24	0.9860	Competitive
**d**	3‐(4‐bromophenyl) isoxazole	13.15 ± 0.15	0.9992	14.48 ± 1.30	0.9895	Competitive
**e**	3‐(4‐fluorophenyl) isoxazole	35.25 ± 1.44	0.9765	37.39 ± 3.32	0.9895	Competitive
**f**	5‐(4‐fluorophenyl) isoxazole	47.22 ± 1.83	0.9885	38.20 ± 3.36	0.9896	Competitive
**g**	5‐(4‐chlorophenyl) isoxazole	43.26 ± 2.49	0.9805	30.71 ± 2.83	0.9884	Competitive
**h**	5‐(4‐methoxyphenyl) isoxazole	51.91 ± 2.23	0.9866	35.94 ± 3.56	0.9865	Competitive
**EPR**	Epalrestat	79.76 ± 5.08	0.9784	232.70 ± 15.51	0.9874	Noncompetitive

*The test results were indicated as mean ± standard error of mean.

Also, the *K_I_* values of the examined compounds (a–h) were 89.51, 12.90, 12.13, 14.48, 37.39, 38.20, 30.71, and 35.94 nM respectively, which were lower than the *K_I_* values of EPR (*K_I_* = 232.70 nM). The importance of substitution at the isoxazole group's 3rd and 5th positions for ALR2 inhibitory efficacy was demonstrated by the experimental results. The isoxazole moiety's para position was modified to accommodate a nitro phenyl substituent, which significantly increased the ALR2 inhibitory action (compound c**;** *K*_I_ = 12.13 nM). However, attachment of different groups such as 4‐chlorophenyl, 4‐nitrophenyl, 4‐bromophenyl, 4‐fluorophenyl, and 4‐methoxyphenyl to the 3rd and 5th positions of isoxazole (compound a, *K*_I_ = 89.51 nM) resulted in a significant increase in *K*_I_ value and a change in the type of inhibition (compound a was found to be a noncompetitive ALR2 inhibitor, while compounds b–h were found to be competitive ALR2 inhibitors).

The 4‐chlorophenyl group at position 3rd of the isoxazole ring (*K*_I_ = 12.90 nM for compound b) inhibited the ALR2 enzyme three times more potently than at position 5th of the isoxazole ring (*K*I = 30.71 nM for compound g). Because of its interactions with the active site of the enzyme and its spatial orientation, the 4‐chlorophenyl group located at the 3rd position of the isoxazole ring probably exhibits higher inhibition of the ALR2 enzyme. The substituent's 3rd‐position placement might promote more effective binding through hydrophobic contacts, hydrogen bonds, or other non‐covalent forces. On the other hand, the 5th position might not offer as favorable an interaction, reducing the binding affinity and, consequently, the inhibitory potency. Around a threefold variation indicates that the third location is more in line with the enzyme's pharmacophore criteria for efficient inhibition.

In addition, the inhibitory effects on the ALR2 enzyme were nearly same for the 4‐fluorophenyl linked to the isoxazole ring at position 3rd (*K*_I_ = 37.39 nM for compound e) and 5th (*K*_I_ = 38.20 nM for compound f). This suggests that the fluorine atom's influence on binding affinity at both positions is similar, potentially due to its small size and electronegativity, which may result in comparable interactions with the enzyme's active site regardless of its position on the isoxazole ring.

The 4‐methoxyphenyl group attached to the isoxazole structure at position 5 (*K*_I_ = 35. 94 nM for compound h) likely inhibits the ALR2 enzyme more effectively than the 4‐fluorophenyl group (*K*_I_ = 37.39 nM for compound e and *K*_I_ = 37.39 nM for compound f) due to differences in electronic and steric properties. The methoxy group (─OCH₃) is an electron‐donating group, which can enhance the interaction with the enzyme's active site by increasing the electron density of the phenyl ring, potentially improving binding affinity through stronger hydrogen bonding or other electrostatic interactions. In contrast, the fluorine atom is more electronegative and smaller, which might result in weaker or less favorable interactions with the enzyme, leading to lower inhibitory potency. In addition, the bulkier nature of the methoxy group compared to fluorine could allow for better hydrophobic interactions in the active site, further contributing to the stronger inhibition.

On the other hand, the 4‐bromophenyl group attached to the 3rd position of the isoxazole ring (*K*_I_ = 14.48 nM for compound d) exhibits an inhibitory effect similar to that of the 4‐chlorophenyl group (*K*_I_ = 12.90 nM for compound b) because bromine and chlorine have similar chemical properties. Both bromine and chlorine are halogens, and while bromine is larger and slightly more polarizable than chlorine, their electronegativity and ability to participate in halogen bonding or hydrophobic interactions are comparable. At the 3rd position, both substituents likely form similar interactions with the ALR2 enzyme's active site, such as van der Waals forces or hydrophobic interactions, resulting in similar inhibitory effects. The slight difference in size between bromine and chlorine may not significantly affect the overall binding affinity, leading to nearly the same inhibitory potency.

Several substances may have an inhibiting effect on ALR2, as documented in the literature. In this regard (Yapar et al. [78]), the ALR2 enzyme was tested for its inhibitory effects on newly synthesized bis‐hydrazone compounds (GY1‐12) with an isovanillin moiety. According to the study results, each of the newly discovered bis‐hydrazones showed activity as ALR2 inhibitors at nanomolar concentrations, with IC_50_ and *K*_I_ values ranging from 12.55 to 35.04 nM and 13.38 to 88.21 nM, respectively. In another study, Demir et al. [37] stated that an ALR2 enzyme obtained from sheep liver was used to test a number of nonsteroidal anti‐inflammatory medications, such as etofenamate, meloxicam, diclofenac, and tenoxicam. The researchers discovered that these nonsteroidal anti‐inflammatory medications had advantageous inhibitory effects on ALR2, with *K*_I_ constants for ALR2 ranging from 3.36 ± 1.08 µM to 17.68 ± 3.39 mM. In another study, Novel molecules based on thiazoles were synthesized by Sever et al. [79]. When compared to quercetin (*K*_I_, 7.025 ± 1.780 µM and IC_50_, 3.233 µM), the compounds have been discovered as possible ALR2Is with *K*_I_ and IC_50_ values in the range of 0.018 ± 0.005–3.746 ± 1.321 µM and 0.983–3.330 µM, respectively. In 2006, Racowitz et al. [80] conducted a study in which they synthesized several 5‐benzyl‐2,4‐thiazolidinediones. The compounds (IC_50_, 1.01–78.9 µM) demonstrated an ALR2 inhibiting action in comparison to reference substances such sorbinil (IC_50_ 3.42 ± 0.02 µM) and ponalrestat (IC_50_, 80 nM). In another study, in 2024, Kaya et al. [81] synthesized a series of imidazo[1,2‐a]pyridine‐based 1,3,4‐thiadiazole derivatives and the ALR2 enzyme was investigated for its inhibitory effects. According to the results they obtained, the novel derivatives (8a–k) demonstrated potential inhibitory activity, with *K*_I_ values covering the following ranges: from 23.47 ± 2.40 to 139.60 ± 13.33 nM for ALR2.

### 3.2 In Silico Study

The molecular docking analyses on ALR2 revealed substantial insights into the binding affinity and interaction characteristics of the isoxazole and its derivatives (a–h) against this enzyme. A systematic evaluation was conducted on these compounds' selectivity and potency. To validate the predictive capability and reliability of the docking procedure, pose‐retrieval experiments were performed using the co‐crystallized ligand, EPR, for ALR2 (PDB ID: 4JIR). By aligning the docked EPR molecule with their experimentally derived structure, we achieved a root‐mean‐square deviation (RMSD) value below 1.0 Å. Such low RMSD values underscore the robustness of the docking approach, confirming that the ligand‐receptor interactions observed here are highly accurate and reproducible, thus ensuring reliable conclusions for the ligand binding pose. The structural characteristics of ALR2's binding pocket are defined by an aromatic gorge that leads to a catalytic triad comprising serine, histidine, and glutamate. This arrangement allows selective ligands to engage in specific aromatic and hydrogen bonding interactions.

**FIGURE 6:**
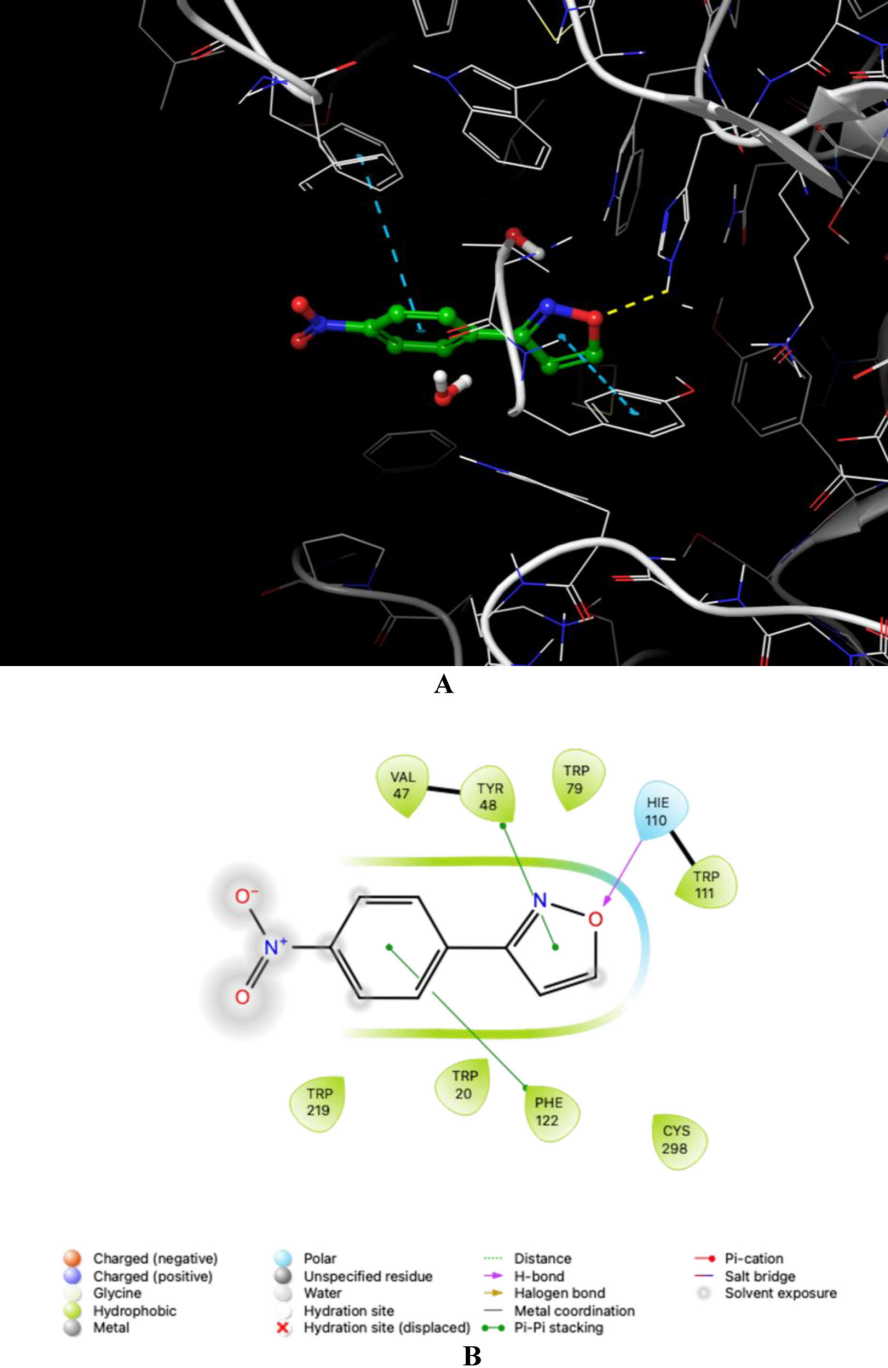
The molecular docking analysis conducted on ALR2, represented by PDB ID 4JIR, with 3‐(4‐nitrophenyl) isoxazole (c). Panel A presents the 3D binding conformation of compound c within the active site of 4JIR, illustrating the spatial orientation and fit within the catalytic pocket. Fundamental binding interactions are indicated, with yellow dashed lines denoting hydrogen bonds and blue dashed lines highlighting π–π stacking interactions with aromatic residues. The 2D interaction map in Panel B further clarifies these binding interactions by depicting specific residues involved in hydrogen bonding and other non‐covalent interactions.

Compound c demonstrated a high affinity for ALR2, exhibiting significant π–π stacking interactions with the Tyr48 and Phe122 residues lining the aromatic gorge. This stacking interaction aligns the ligand within the active site. It enhances binding stability, indicating that aromatic interactions are essential for the structural congruency and strength of the enzyme‐ligand complex. In addition, the pyrazole ring in compound c established a hydrogen bond with His110, with a distance of 2.27 Å, further strengthening the enzyme‐ligand complex. This precise interaction improved the docking score to −5.59 kcal/mol, aligning with previous findings that underscore the importance of aromatic stacking for the binding stability of ALR2I (Figure 6).

The ADME predictions for each ligand yielded valuable insights into their pharmacokinetic properties, which are pivotal in determining potential drug viability. Ligands displaying high binding affinities, particularly those with optimal hydrophobic and hydrogen bonding interactions within the enzyme active sites, also demonstrated favorable absorption and distribution profiles. This trend suggests that these ligands are structurally compatible with physiological transport and distribution mechanisms, reinforcing their suitability as potential inhibitors. In contrast, ligands possessing larger or highly polar functional groups presented reduced absorption potential, a limitation that may hinder bioavailability and restrict their effectiveness in systemic applications.

These findings emphasize the influence of specific molecular characteristics, such as functional group polarity, size, and spatial orientation, on the pharmacokinetic behavior of each ligand. Consequently, the ADME profile underscores the importance of strategic ligand design in optimizing drug‐like properties and enhancing therapeutic potential. The molecular docking results further reveal the unique binding preferences of ALR2, shaped by distinct structural configurations within their active sites. Notably, among the isoxazole derivatives, 3‐(4‐nitrophenyl) isoxazole (c) emerged as a standout candidate, exhibiting robust binding affinities and favorable interaction profiles with key residues. These promising interactions suggest a high potential for these derivatives to serve as lead compounds, meriting further investigation through experimental validation (Table 2).

**TABLE 2:** ADME‐related parameters of the isoxazole and its derivatives (a–h) and reference drug epalrestat.

Parameters	Compound ID								
	a	b	c	d	e	f	g	h	EPR
MW	69.06	179.61	190.16	224.06	163.15	163.15	179.61	175.19	319.39
QPlogPoct	3.17	7.40	9.09	7.52	6.97	6.94	7.38	8.00	15.09
QPlogPw	3.27	4.17	5.55	4.18	4.19	4.22	4.20	4.67	8.07
QPlogPo/w	0.80	2.70	1.13	2.74	2.26	2.27	2.70	2.77	3.65
QPlogS	−0.33	−2.72	−2.04	−2.93	−2.20	−2.25	−2.78	−2.27	−4.51
QPlogHERG	−2.79	−4.24	−4.33	−4.28	−4.16	−4.23	−4.31	−4.28	−3.36
QPlogBB	0.10	0.33	−0.73	0.34	0.27	0.25	0.30	0.65	−0.95
QPlogKp	−2.16	−1.58	−3.32	−1.58	−1.55	−1.59	−1.62	−1.55	−2.88
QPlogKhsa	−0.90	−0.87	−0.31	−0.61	−0.17	−0.16	−0.77	−0.23	0.10
Human oral absorption %	87.88	100.00	79.82	100.00	100.00	100.00	100.00	100.00	84.69
PSA	27.71	25.76	70.78	25.76	25.76	26.44	26.44	34.64	88.94
Rule of five	0	0	0	0	0	0	0	0	0
Rule of three	0	0	0	0	0	0	0	0	0

Through these extensive computational assessments, the molecular docking and pharmacokinetic predictions collectively contribute valuable data on the binding efficacy and potential drug viability of these derivatives against ALR2, paving the way for future in vitro validations.

## 4 Conclusions

Testing isoxazole derivatives as ALR2Is, followed by docking studies, represents a pioneering approach. It allows for the rational design of new compounds with improved inhibitory activity and pharmacological profiles. Such studies' findings may result in the development of novel therapeutic medicines for the treatment of diabetes complications. The isoxazole ring, due to its ability to form hydrogen bonds and interact with hydrophobic pockets in the enzyme, shows promise in modulating aldose reductase activity effectively. The combination of experimental inhibition assays and computational docking studies creates a comprehensive strategy for evaluating isoxazole derivatives' efficacy as aldose reductase inhibitors, paving the way for novel drug candidates in the management of diabetes‐related conditions. Within the scope of this research, we revealed the inhibition effect of isoxazole and some of its derivatives (a–h) on the ALR2 enzyme. Consequently, an assessment was conducted to determine the inhibitory ability of these drugs against ALR2, the primary enzyme of the polyol pathway implicated in diabetes mellitus. Our results show that these chemicals have a variety of inhibitory effects, and their effects on ALR2 are particularly significant. Notably, all isoxazoles demonstrated a considerable inhibition of ALR2; their efficacy was superior to that of the reference drug EPR (*K*_I_ value of 232.70 ± 15.51 nM), with values ranging from 12.13 ± 1.24 to 89.51 ± 1.24 nM. It was found that compound c (*K*_I_ value of 12.13 ± 1.24 nM) was a more effective inhibitor of ALR2 enzyme activity than EPR among the substances whose effects were investigated. It is anticipated that our research will contribute to the creation of new ALR2 inhibitors through studies on inhibition and molecular docking. We also hope that these compounds may become better treatment agents for use in the treatment of diabetes mellitus as a result of further studies.

## Author Contributions

**Ahmet Esat Göner**: writing – original draft, validation, investigation, formal analysis. **Hatice Esra Duran**: writing – original draft, validation, methodology, investigation, funding acquisition, formal analysis, conceptualization.

## Conflicts of Interest

The authors declare no conflicts of interest.

## Data Availability

The data supporting the study's conclusions will be made available by the corresponding author upon reasonable request.
